# Phytochemical Molecules from the Decarboxylation of Gomphrenins in Violet *Gomphrena globosa* L.—Floral Infusions from Functional Food

**DOI:** 10.3390/ijms21228834

**Published:** 2020-11-22

**Authors:** Natalia Drobnicka, Katarzyna Sutor, Agnieszka Kumorkiewicz-Jamro, Aneta Spórna-Kucab, Michał Antonik, Ewa Dziedzic, Tomasz Świergosz, Joanna Ortyl, Sławomir Wybraniec

**Affiliations:** 1 Department of Analytical Chemistry, Faculty of Chemical Engineering and Technology, Cracow University of Technology, Warszawska 24, 31-155 Kraków, Poland; nszmyr@chemia.pk.edu.pl (N.D.); katarzyna.sutor@doktorant.pk.edu.pl (K.S.); agnieszka.kumorkiewicz-jamro@pk.edu.pl (A.K.-J.); aneta.sporna-kucab@pk.edu.pl (A.S.-K.); michal3antonik@gmail.com (M.A.); tomasz.swiergosz@pk.edu.pl (T.Ś.); 2 Department of Horticulture, Faculty of Biotechnology and Horticulture, Hugo Kołłątaj University of Agriculture, 29 Listopada 54, 31-425 Kraków, Poland; ewa.dziedzic@urk.edu.pl; 3 Department of Biotechnology and Physical Chemistry, Faculty of Chemical Engineering and Technology, Cracow University of Technology, Warszawska 24, 31-155 Kraków, Poland; jortyl@pk.edu.pl or; 4 Photo HiTech Ltd., Bobrzyńskiego 14, 30-348 Cracow, Poland

**Keywords:** functional food, gomphrenins, decarboxylation, infusion, LCMS-IT-TOF

## Abstract

Herein, the generation of decarboxylated derivatives of gomphrenin pigments exhibiting potential health-promoting properties and the kinetics of their extraction during tea brewing from the purple flowers of *Gomphrena globosa* L. in aqueous and aqueous citric acid solutions were investigated. Time-dependent concentration monitoring of natural gomphrenins and their tentative identification was carried out by LC-DAD-ESI-MS/MS. The high content of acylated gomphrenins and their principal decarboxylation products, 2-, 15-, 17-decarboxy-gomphrenins, along with minor levels of their bidecarboxylated derivatives, were reported in the infusions. The identification was supported by the determination of molecular formulas of the extracted pigments by liquid chromatography coupled with high-resolution mass spectrometry (LCMS-IT-TOF). The influence of plant matrix on gomphrenins’ stability and generation of their derivatives, including the extraction kinetics, was determined by studying the concentration profiles in the primary and diluted infusions. Isolated and purified acylated gomphrenins from the same plant material were used for the preliminary determination of their decarboxylated derivatives. The acylated gomphrenins were found to be more stable than nonacylated ones. Citric acid addition had a degradative influence on natural gomphrenins mainly during the longer tea brewing process (above 15 min); however, the presence of plant matrix significantly increased the stability for betacyanins’ identification.

## 1. Introduction

*Gomphrena globosa* L. commonly known as globe amaranth is an annual herbaceous, edible plant belonging to the family of Amaranthaceae. It is widely cultivated in China and the tropical regions of Central America. *G. globosa* inflorescences occur basically in three varieties: white, red, and violet. They are utilized in traditional Chinese medicine in the preparation of cough syrups to treat respiratory system diseases. Dried gomphrena blossoms are characterized by unique fragrance and exhibit numerous pro-health properties. They are commonly used for the preparation of tea infusions. Furthermore, globe amaranth flowers are sold on a commercial scale as tisanes and used as a popular ingredient in blooming teas which are commonly known as flowering tea [1]. Nowadays, the number of people suffering from civilization diseases such as diabetes, hypertension, and cancer, is increasing. Therefore, natural products comprising biologically active compounds attract much attention [2]. These products implemented into a daily diet may play an important role in the prevention and treatment of many health problems [3]. The violet *G. globosa* inflorescences are a precious source of many antioxidants involving betalains. These compounds are proven to exhibit numerous pro-health properties, owing to the high content of hydroxyl groups that participate in free radical scavenging [3,4,5,6,7]. Their antihemorrhage, antimicrobial, antiinflammatory activity, and analgesic effects have been reported [8,9,10,11,12,13]. Betalains are vacuolar, natural plant pigments. They are present in plants belonging to the Caryophyllales order [14,15]. It is worth noting that betacyanins acylated by hydroxycinnamic acid derivatives are present in gomphrena flowers at relatively high levels. To simplify, they are frequently denominated as gomphrenins. Accordingly, nonacylated gomphrenin I is a common name for betanidin-6-*O*-*β*-glucoside, whereas gomphrenin II and III are betanidin-6-*O*-(6′-*O*-trans-4-coumaroyl)-*β*-glucoside and betanidin-6-*O*-(6′-*O*-trans-feruloyl)-*β*- -glucoside, respectively [16,17,18]. Their chemical structures have been completely elucidated by nuclear magnetic resonance and mass spectrometry [19,20]. The structure of gomphrenin IV has been tentatively elucidated as a betanidin-6-*O*-(6′-*O*-sinapoyl)-*β*-glucoside because of its coelution with gomphrenin III. Betanidin 6-*O*-glucosides are very rare compounds and hitherto, they have been identified only in *G. globosa* inflorescences, *B. alba* L. fruits and its variety *B. rubra* L. (the main source of nonacylated gomphrenin I) as well as in *B. glabra* bracts [19,20,21,22,23,24,25,26]. Previous reports indicated that decarboxylated betacyanin derivatives are formed in preparations subjected to thermal processing as a result of betalains’ sensitivity to elevated temperatures [21,24,27,28,29,30,31,32]. Thermal decarboxylation may occur at different degrees. In general, 2-decarboxy- and 17-decarboxy-betacyanins, which exist in two diastereomeric forms, are formed in the greatest amounts. Furthermore, 15-decarboxy-betacyanins with the lost chiral center at the C-15 carbon are observed, therefore, there are no additional diastereomers of these derivatives. Frequently, after prolonged heating, bidecarboxy- and even tridecarboxy-betacyanins can be formed as well. Decarboxylated betacyanins are less polar than the initial forms of betacyanins, therefore, their retention on reversed-phase in HPLC is greater [21,24]. Despite a few phytochemical studies concerning the presence of phenolic compounds in *G. globosa* flowers, there is a lack of reports referring to the comprehensive extraction of these relevant compounds during thermal processing close to tea brewing conditions [7,28]. For that reason, our study aimed to evaluate the kinetics of betacyanins’ extraction in tea infusion prepared from *G. globosa* flowers and to determine betacyanin degradation products. Research on the influence of citric acid addition and matrix presence on the stability of the compounds during the preparation of *G. globosa* infusions as well as the comparative experiments with single purified gomphrenins derived from *G. globosa* flowers was also performed.

## 2. Results and Discussion

**Figure 1:**
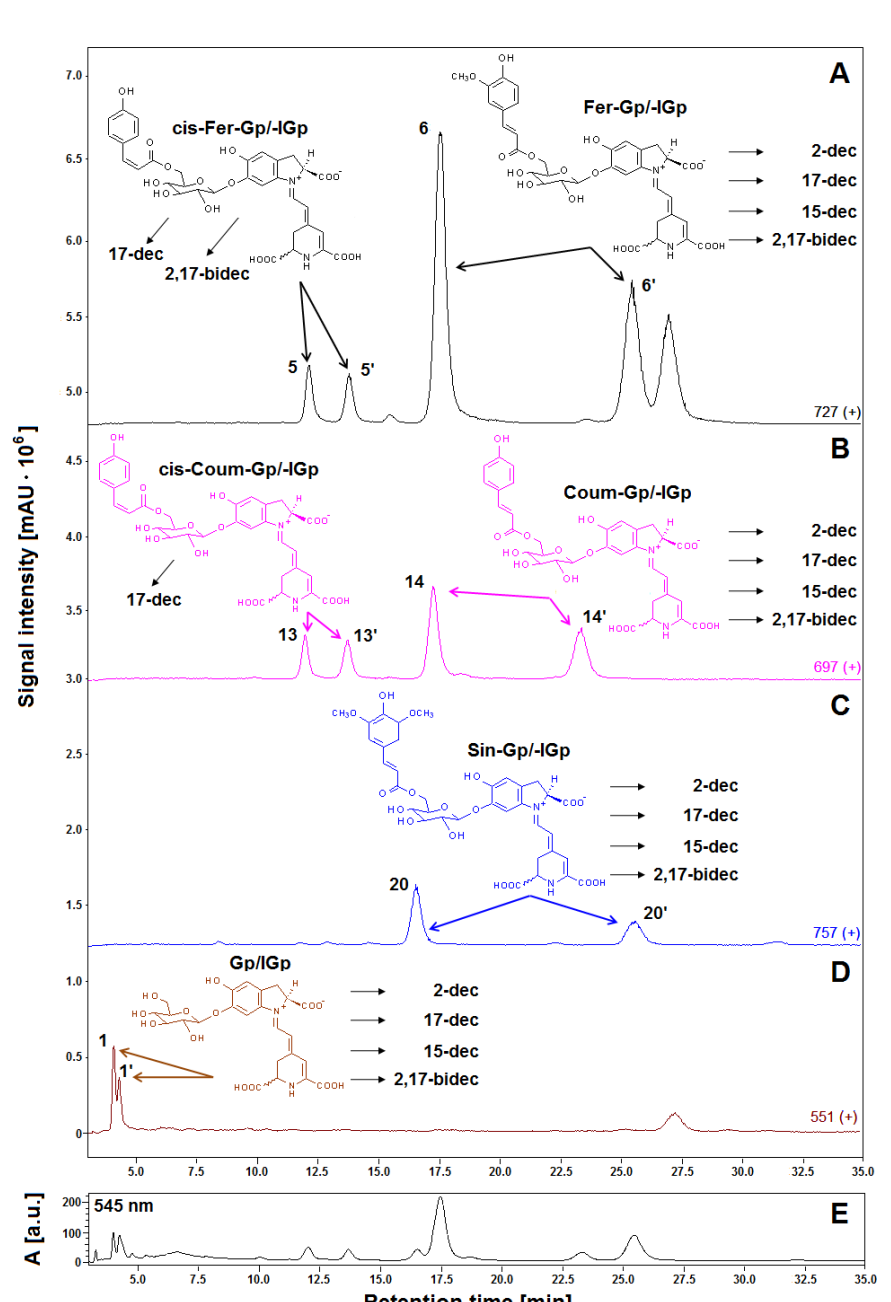
LCMS gomphrenin profiles (**A**–**D**) recorded in selected ion monitoring (SIM) mode of the freeze-dried representative extract obtained from purple *G. globosa* flowers and chemical structures of natural gomphrenins present in the extract which undergo decarboxylation during the tea brewing process leading to obtaining products which had their abbreviations placed. The LC-DAD fingerprint (λ 545 nm) of the betacyanin extract is also depicted for an indication of the pigments (**E**).

**Figure 2:**
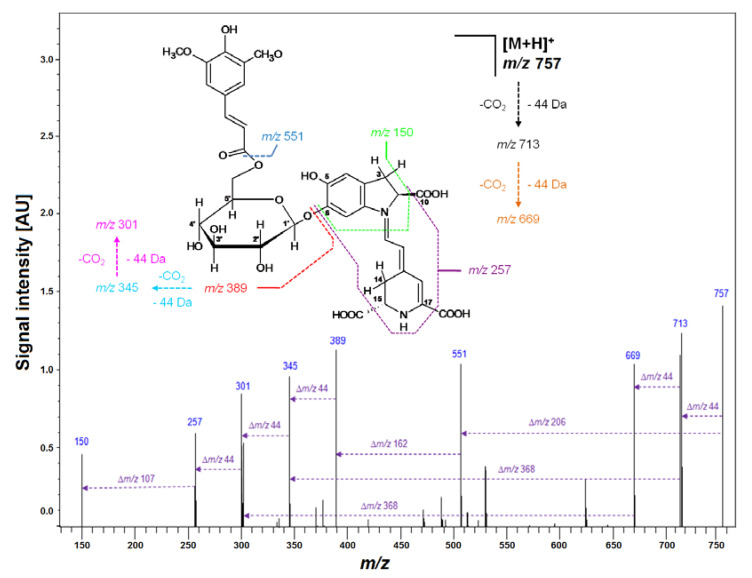
Fragmentation pattern obtained by LCMS-IT-TOF for purified Sin-Gp **20** isolated from purple *G. globosa* flowers.

Previous studies revealed a typical primary betacyanin profile in the extract of violet *G. globosa* flowers [12,22]. Chromatograms recorded in selected ion monitoring mode (SIM), and DAD chromatogram registered at 545 nm, obtained for a lyophilized sample of *G. globosa* extract not subjected to heating are depicted in Figure 1. Additionally, chemical patterns of natural gomphrenins and abbreviation names of the observed decarboxylated derivatives are presented. The presence of sinapoyled diastereomers **20/20′** has never been confirmed in natural extracts of *G. globose*, except for low-resolution mass spectrometry (Table 1) [12,22]; therefore, additional high-resolution measurements and confirmation of the molecular formulas were performed by LCMS-IT-TOF (Figure 2, Table 2).

The dominant presence of the feruloyl-gomphrenin (Fer-Gp) **6**, coumaroyl-gomphrenin (Coum-Gp) **14** and their diastereomers (Fer-IGp) **6′**, (Coum-IGp) **14′** with significantly lower quantities of sinapoyl-gomphrenin/-isogomphrenin (Sin-Gp/-IGp) **20/20′** and nonacylated gomphrenin I/isogomphrenin I (Gp/IGp) **1/1′** was confirmed. For the aim of simplification, we propose to refer to the acylated pigments as respective “hydroxycinnamoyled gomphrenins” as well as to their abbreviations (Table 1) instead of selected “gomphrenins II, III, and IV”. Accordingly, nonacylated “gomphrenin I” will be referred to as “gomphrenin”. In the previous study [12], additional cis-isomers of coumaroyl-gomphrenin (cis-Coum-Gp/-IGp) **13/13′** and feruloyl-gomphrenin (cis-Fer-Gp/-IGp) **5/5′** were tentatively detected.

**Figure 3:**
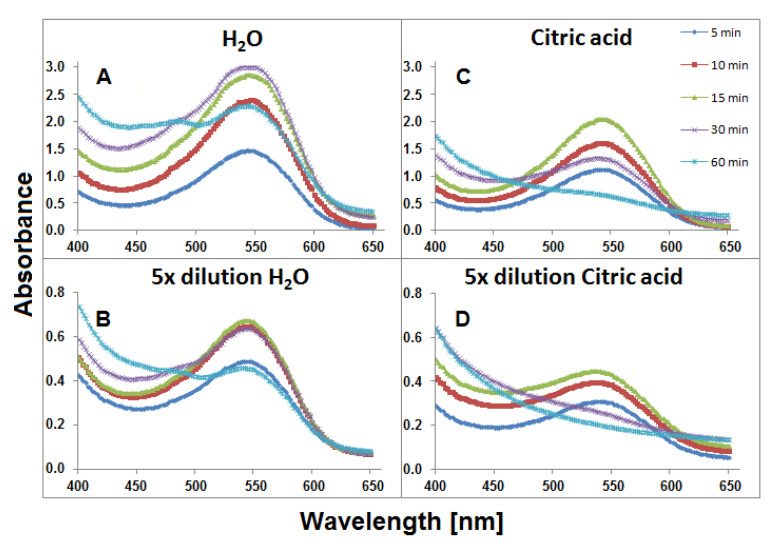
Visible spectra of aqueous (**A** and **B**) and citric acid (**C** and **D**) infusions of *G. globosa* flowers obtained within 60 min of tea brewing at 90 °C.

For 0.94 g or 0.19 g weighted portions of *G. globosa* flowers which were brewing in 6 mL of water for 60 min, two series of solutions were obtained, nondiluted, and fivefold diluted, respectively. The temperature (90 °C) of the tea brewing process was high enough for monitoring changes in the compositions of the resulting mixtures and close to home tea brewing conditions. The spectra of the visible range of the obtained extract samples collected during heat processing are shown in Figure 3. Higher absorbance values were observed for the samples which were extracted in aqueous solutions (Figure 3A,B) in comparison to aqueous citric acid solutions. It indicates mild extraction conditions of gomphrenins in water without citric acid addition. A stronger influence of citric acid addition on the stability of these compounds can be observed in Figure 3C,D. After 60 min, for undiluted samples, the absorption maximum was not clearly observed and there was no absorption band for diluted samples after 30 min.

**Figure 4:**
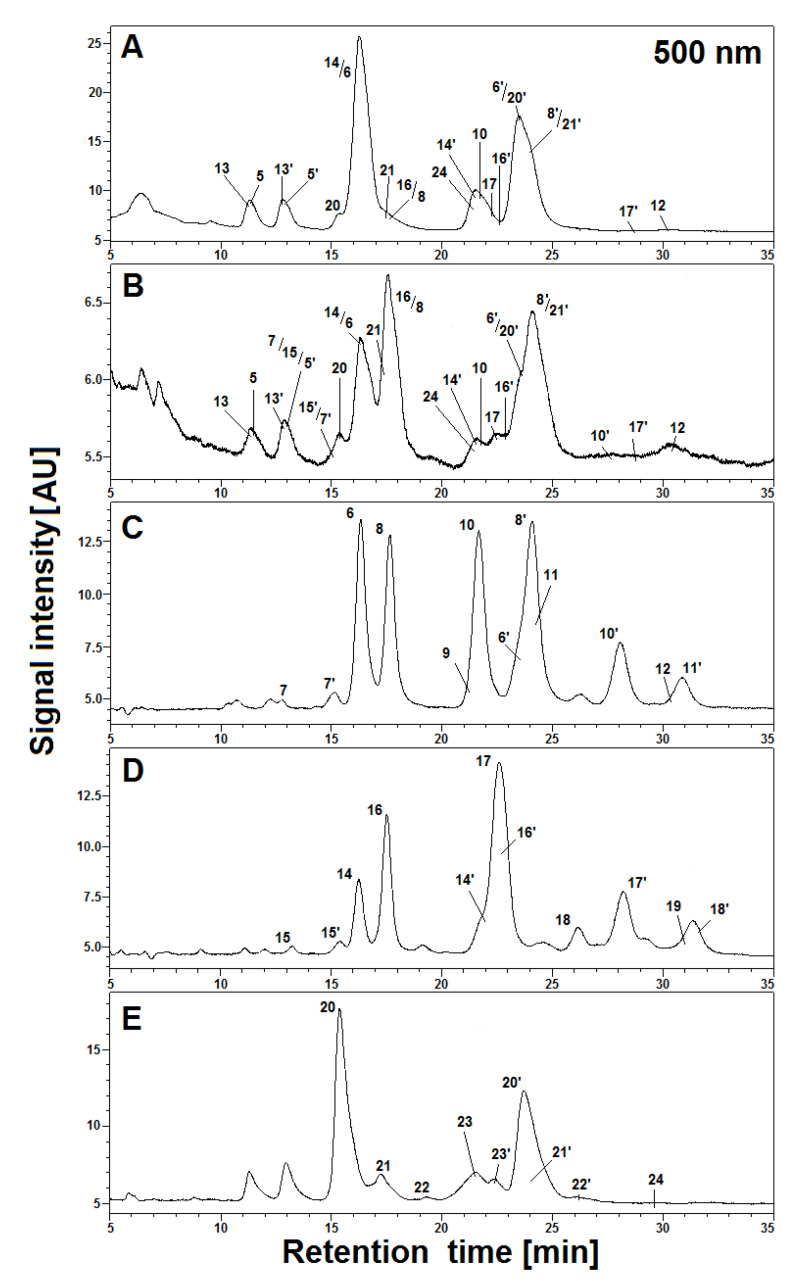
HPLC profiles (λ 500 nm) of acylated gomphrenin and gomphrenin-based derivatives (**A** and **B**) in 5× diluted tea infusions of purple *G. globosa* flowers obtained in aqueous (**A**) and citric acid solutions (**B**) after 30 min of brewing at 90 °C, as well as referential HPLC profiles of purified feruloylated (**C**), coumaroylated (**D**), and sinapoylated (**E**) gomphrenin derivatives obtained after 20 min (**C** and **D**) and 10 min (**E**) of heating at 90 °C in citric acid solutions.

This resulted from the lower impact of the plant matrix which presumably supported the stability of the pigments. The slight absorption maximum at approximately 480 nm is observed in Figure 3A after 60 min. It indicates the presence of other conversion products, presumably oxidized, of natural gomphrenins. Decarboxylated degradation products of natural gomphrenins formed during the tea brewing process in aqueous and citric acid solutions are listed in Table 1. All the detected degradation products of the pigments were less polar than their corresponding precursors. For the most prominent degradation products of acylated gomphrenins, additional confirmation was obtained by LCMS-IT-TOF analyses (Table 2). A series of selected chromatograms obtained for the processed flowers in the experiments as well as for selected purified and heated pigments are depicted in Figure 4.

### 2.1. Extraction of Gomphrenins during Tea Brewing of G. globosa in Aqueous Solutions

**Figure 5:**
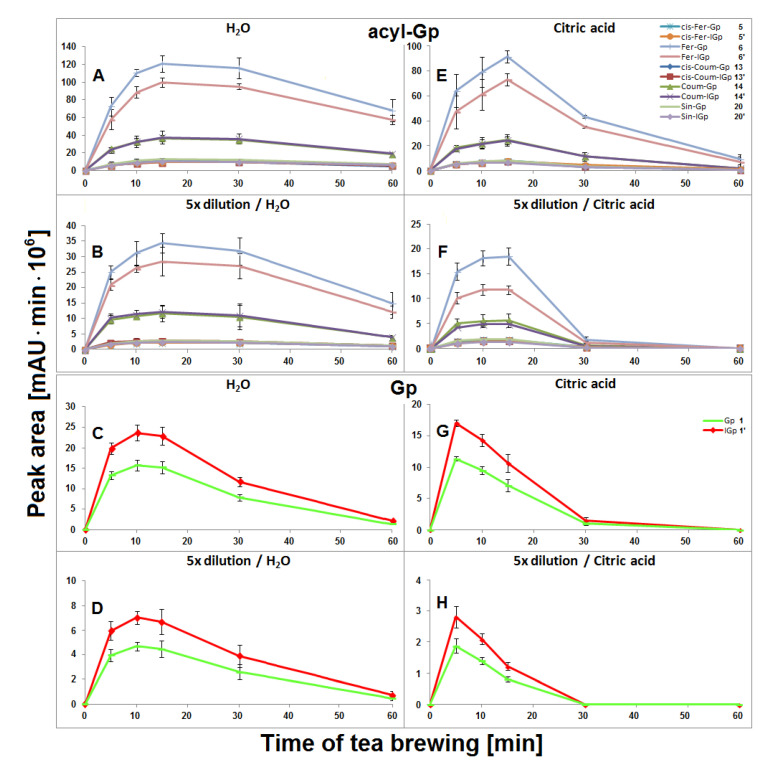
Time-dependent concentration profiles of gomphrenins and acylated gomphrenins obtained after brewing purple *G. globosa* flowers at 90 °C in undiluted (**A**,**C**,**E**,**G**) and 5× diluted (**B**,**D**,**F**,**H**) aqueous and citric acid solutions. Standard deviation values (SD) of nonacylated pigments **1/1′** in citric acid solutions were multiplied by a factor of 3 and 2 for undiluted (**G**) and diluted samples (**H**), respectively.

The highest concentration of all acylated gomphrenins in undiluted and fivefold diluted samples were obtained after 15 min of extraction, but the degradation of substrates was faster for diluted samples, presumably as a result of the diminished stabilizing effect of the matrix in diluted solutions (Figure 5). The matrix effect in aqueous solutions was not clearly observed for Gp/-IGp **1/1′,** but these betacyanins were, in general, less stable during tea brewing than the acylated ones. The greatest signal intensity for Gp/-IGp **1/1′** was observed after 10 min of thermal treatment.

### 2.2. Generation and Identification of 17-Decarboxylated Derivatives of Gomphrenins in Aqueous Solutions

**Figure 6:**
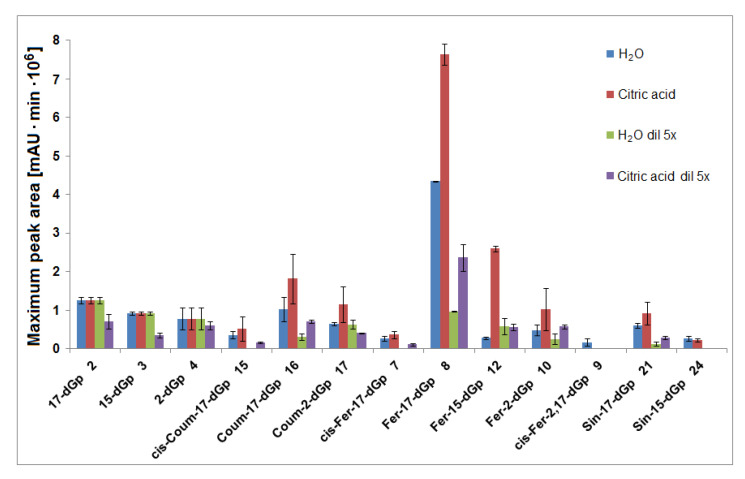
Maxima of time-dependent concentration profiles of decarboxylated gomphrenin derivatives generated in the course of 60 min tea brewing experiments in aqueous and citric acid solutions at 90 °C.

Interpretation of the LC-DAD and LC-MS spectra obtained in the HPLC gradient System 5 revealed that the main products appeared to be mono-decarboxylated derivatives for all gomphrenins due to the loss of CO_2_ from the corresponding precursors (Figure 4 and Figure 6). Based on previous studies [24], a group of distinct chromatographic peaks with characteristic absorption maxima at λ_max_ 507, 515, 512, and 517 nm, influenced by the bathochromic effect of the acyl substituents, was attributed to 17-decarboxylated derivatives of nonacylated gomphrenins (17-dGp/-dIGp) **2/2′** as well as feruloylated (Fer-17-dGp/-dIGp) **8/8′**, coumaroylated (Coum-17-dGp/-dIGp) **14/14′,** and sinapoylated gomphrenins (Sin-17-dGp/-dIGp) **21/21′**, respectively. Similarly, cis-feruloyl-17-decarboxy-gomphrenin/-isogomphrenin (cis-Fer-17-dGp/-dIGp) **7/7′** and cis-coumaroyl-17-decarboxy-gomphrenin/-isogomphrenin (cis-Coum-17-dGp/-dIGp) **15/15′** were tentatively detected (Table 1) only in undiluted samples due to very low signal intensities (Figure 6).

**Figure 7:**
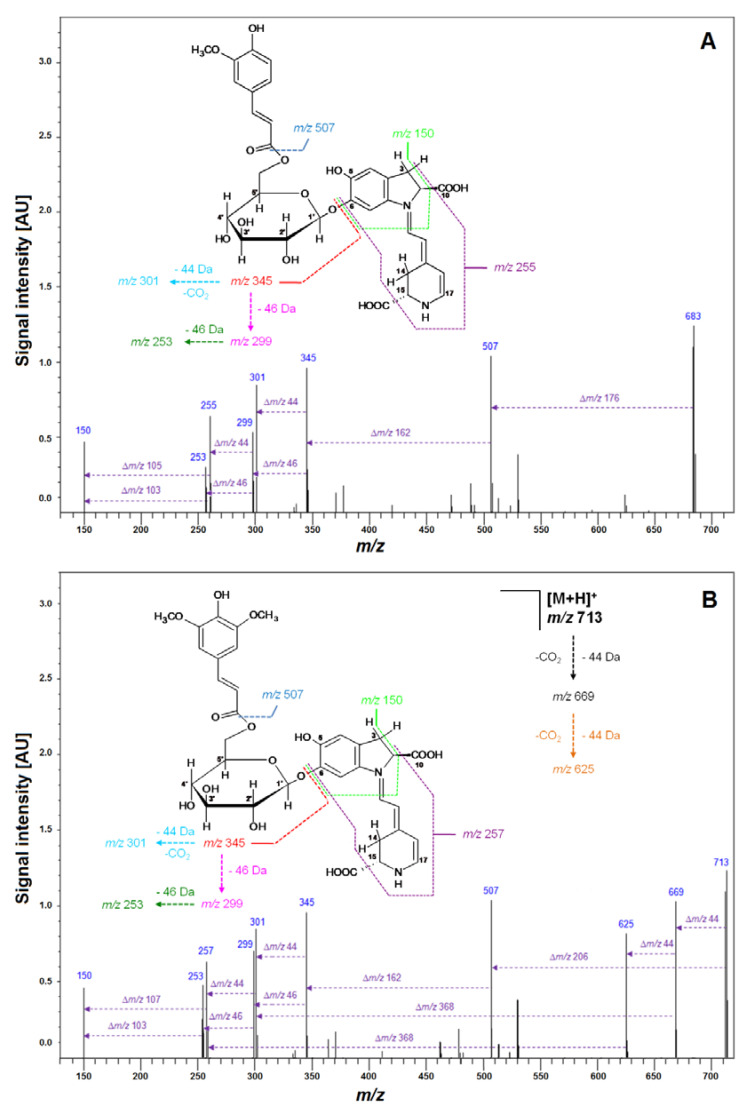
Fragmentation patterns obtained by LCMS-IT-TOF for Fer-17-dGp **8** (**A**) and Sin-17-dGp **21** (**B**) formed in the course of heating at 90 °C for 20 min of isolated Fer-Gp **6** and Sin-Gp **20** from *G. globosa* flowers.

Subsequently, LCMS-IT-TOF analyses yielding *m/z* 683.2081 (C_33_H_34_N_2_O_14_, calculated m/z: 683.2083) confirmed the molecular formula of Fer-17-dGp **8** (Table 2). The observed fragmentation pathway in the MS^2^ mode afforded a signal at *m/z* 507 (Table 2), indicating detachment of a feruloyl moiety at the glucosyl ring of mono-decarboxylated gomphrenin (683 − 507 = 176 Da). Other daughter ions of Fer-17-dGp **8** detected at *m/z* 345 and 301 were assigned to mono- and bi-decarboxylated betanidin as a result of further deglycosylation (507 − 345 = 162 Da) and decarboxylation at carbon C-2 or C-15 (345 − 301 = 44 Da), respectively. The full fragmentation pattern is depicted in Figure 7. Likewise, HRMS determination of the molecular formula of C_32_H_32_N_2_O_13_ for the precursor ion of Coum-17-dGp **16** at *m/z* 653 supported the presence of a decarboxylated coumaroylated gomphrenin (determined *m/z* 653.1990; calculated *m/z* 653.1977). The fragmentation pattern of Coum-17-dGp **16** was analogous to that of Fer-17-dGp **8** and allowed for detection of the detachment of coumaroyl moiety (653 − 507 = 146 Da) from the glucosyl ring of mono-decarboxylated gomphrenin as well as the fragmentation ions at *m/z* 345 and 301. Fragmentation ions at *m/z* 507 and 345 were observed for cis-Fer- and cis-Coum-17-dGp (**7** and **15**). Sin-17-dGp **21** exhibited a precursor ion [M + H] + at *m/z* 713 and its molecular formula, C_34_H_36_N_2_O_15_, was obtained in HRMS analyses (determined *m/z* 713.2205; calculated *m/z* 713.2188). Its fragmentation pathway was analogous to the previously described Fer-17-dGp **8** as well as Coum-17-dGp **16,** and it was presented in Figure 4. Detection of a fragmentation ion at *m/z* 507 supported the presence of Sin-17-dGp **21** (713 − 507 = 206 Da).

### 2.3. Concentration Profiles of 17-Decarboxylated-Gomphrenins in Aqueous Solutions

**Figure 8:**
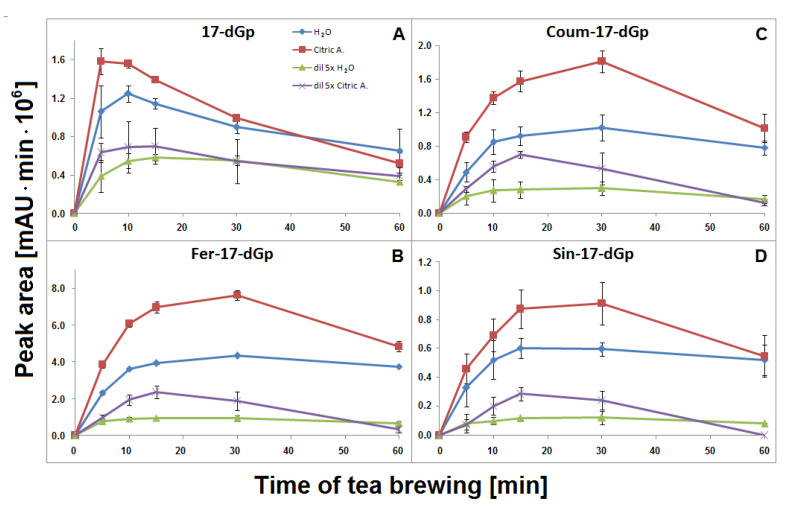
Time-dependent concentration profiles of 17-decarbooxy-derivatives of nonacylated gomphrenin (**A**) and feruloylated, coumaroylated, and sinapoylated gomphrenins (**B**–**D**, respectively) generated during brewing of purple *G. globosa* flowers at 90 °C in undiluted and 5× diluted aqueous and citric acid solutions. These data represent time-dependent concentration of generated of 2-, 15-, and 2,17-decarboxy-gomphrenins. For clarity, calculated SD values for aqueous solutions were divided by a factor of 2 for 17-dGp (**A**) in undiluted aqueous solution of citric acid and diluted aqueous solutions and by a factor of 2 and 5 for Coum-17-dGp in undiluted aqueous and citric acid samples, respectively (**C**).

**Figure 9:**
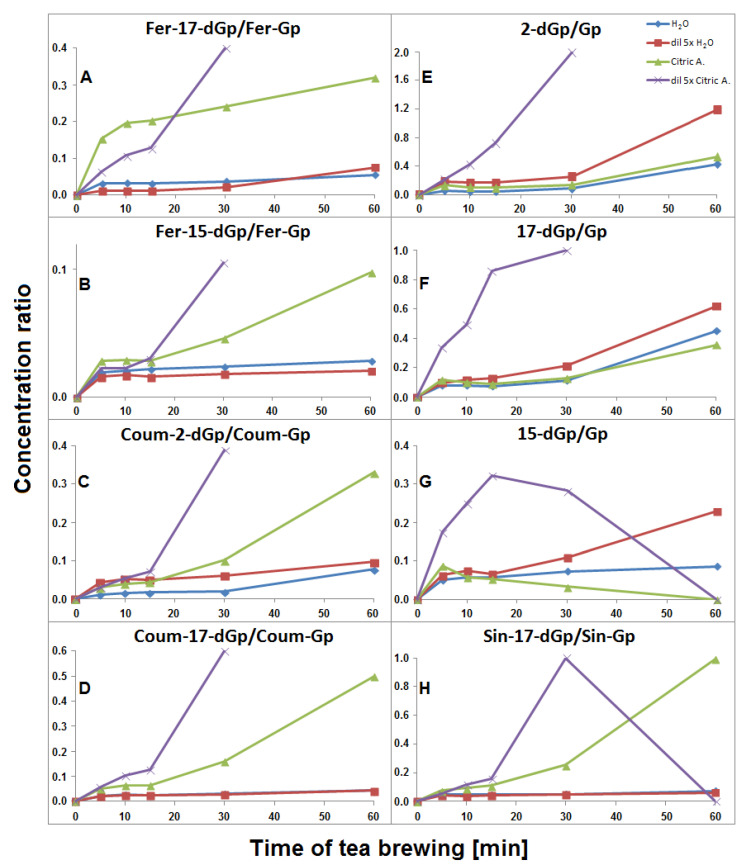
Time-dependent concentration ratio CR of generated feruloylated (**A** and **B**), coumaroylated (**C** and **D**) and sinapoylated (**H**) as well as non-acylated (**E**–**G**) decarboxylated derivatives to their corresponding natural gomphrenins during *G. globosa* thermal processing at 90 °C. The ratios obtained for diluted citric acid samples after 60 min are not shown for most compounds due to a very high degradation rate of natural gomphrenins in citric acid solutions.

The time-dependent concentration profiles for 17-dGp **2** as well as acylated Fer-17-dGp **8**, Coum-17-dGp **16**, and Sin-17-dGp **21** in all the samples during tea brewing are shown in Figure 8A–D. The maximal concentrations for acylated derivatives **8**, **16**, and **21** were evidently shifted to 30 min in comparison to their parent substrates **6, 14**, and **20** (Figure 5), for which the maxima were observed at ca. 15 min of the brewing experiment. This indicates that a continuous decarboxylation effect took place in the substrates Fer-Gp **6** and Coum-Gp **14** as a result of their heating in the presence of relatively high quantities of the inflorescence matrix. This effect is less demonstrated for the diluted samples (Figure 8B,C) presumably as a result of a lower concentration of the pigments and a lower stabilizing effect of the matrix, therefore, faster degradation of the generated pigments was observed. For the Sin-17-dGp **21**, these differences were less pronounced (Figure 8D), most probably because of the much lower concentration of the parent substrate Sin-Gp **20** in the flowers. Concentration ratios (CR) of the resulting Fer-17-dGp **8**, Coum-17-dGp **16**, and Sin-17-dGp **21** to their respective natural gomphrenins slowly increased in the course of heating both in concentrated and diluted samples (Figure 9A,D,H, respectively). For 17-dGp **2**, the concentration maximum was observed at 10–15 min of brewing (Figure 8A). This presumably results from a low concentration of Gp **1** in the flowers, but also from its much lower stability (evidenced in Figure 5C,D). The CR value of 17-dGp **2** to nonacylated gomphrenin **1** (Figure 9F) increased definitely faster than CR for acylated decarboxylated gomphrenins presumably due to lower stability of the initial pigment. For cis-Fer-17-dGp **7** and cis-Coum-17-dGp **15**, the concentration profiles (data not shown) were virtually the same as for Fer-17-dGp **8** and Coum-17-dGp **16**.

### 2.4. Generation and Identification of 2-Decarboxylated Derivatives of Gomphrenins in Aqueous Solutions

Other mono-decarboxylated compound **10** and the pair of **17/17′** were assigned to feruloyl-2-decarboxy-gomphrenin (Fer-2-dGp) and coumaroyl-2-decarboxy-gomphrenin/-isogomphrenin (Coum-2-dGp/-dIGp), respectively. These compounds displayed the characteristic absorption maxima at λ_max_ 537 and 539 nm [24,28,29] and characteristic protonated molecular ions [M + H] + at *m/z* 683 and 653. For Fer-2-dGp **10**, LCMS-IT-TOF analyses yielding *m/z* 683.2075 confirmed the molecular formula of C_32_H_32_N_2_O_13_. The same fragmentation ions as for Fer-17-dGp **8** were observed for Fer-2-dGp **10**, besides the ion at *m/z* 301 that was not detected (Table 2).

### 2.5. Concentration Profiles of 2-Decarboxylated-Gomphrenins in Aqueous Solutions

Fer-2-dGp **10** was generated in the concentrated and diluted samples mainly within the first 10–15 min of tea brewing and most probably underwent following chemical changes (by decarboxylation) during the rest of the process, similarly to Fer-17-dGp **8** (Figure 8B). The diastereomer Fer-2-dIGp **10′** was not observed in the aqueous tea infusion samples. The time-dependent concentration profile of Coum-2-dGp **17** was very similar to the profile obtained for Coum-17-dGp **16** during thermal treatment (Figure 8C). CR values of Coum-2-dGp **17** slowly increased, similarly to Coum-17-dGp **16** (Figure 9C). For gomphrenin, one chromatographic peak, corresponding to 2-decarboxy-gomphrenin/-isogomphrenin **4/4′,** was observed. This signal was characterized by absorption maximum at λ_max_ 533 nm, which is coherent with the previous study [24]. Moreover, these data are close to the results obtained for betanin thermal degradation with 2-decarboxy-betanin/-isobetanin being the products present in heated betanin-rich red beet juice [21,25,26,27,30]. The concentration profile and the CR value of 2-dGp **4** was comparable to 17-dGp **2** (Figure 8A and Figure 9E).

### 2.6. Generation and Identification of 15-Decarboxylated Derivatives of Gomphrenins in Aqueous Solutions

The 15-decarboxy-derivatives, which were formed with a loss of the chiral center at carbon C-15 and exhibited only single chromatographic peaks, were tentatively identified for gomphrenin as well as feruloylated and sinapoylated gomphrenins. 15-decarboxy-gomphrenin (15-dGp) **3** and feruloyl-15-decarboxy-gomphrenin (Fer-15-dGp) **12** displayed the characteristic absorption maxima at λ_max_ 530 nm. LC-MS and HRMS LCMS-IT-TOF spectra and the fragmentation ions were analogous to the data for 17-decarboxy-derivatives. The absorption maxima could not be observed for sinapoyl-15-decarboxy-gomphrenin (Sin-15-dGp) **24** due to low signal intensity.

### 2.7. Concentration Profiles of 15-Decarboxylated-Gomphrenins in Aqueous Solutions

The time-dependent concentration profiles of the acylated derivatives (Fer-15-dGp **12** and Sin-15-dGp **24**) were close to the profiles of 17-decarboxylated derivative 8 (Figure 8B), while the nonacylated derivative **3** level decreased after 10 min, similarly to 17-dGp **2** (Figure 8A). Comparable concentration levels were observed for Fer-15-dGp **12** and Sin-15-dGp **24** in concentrated samples, whereas Sin-15-dGp **24** was not detected in diluted samples (Figure 6). The CR values of Fer-15-dGp **12** were comparable to Fer-17-dGp **8** during tea brewing, whereas the CR ratio of resulting 15-dGp **3** in dilute aqueous samples increased much faster (Figure 9B,G).

### 2.8. Generation of Bi-Decarboxylated Derivatives of Gomphrenins

In the course of tea brewing, one bi-decarboxylated derivative of the native acylated gomphrenin was tentatively detected as cis-feruloyl-2,17-bidecarboxy-gomphrenin (cis-Fer-2,17-dGp) **9**. The concentration profile of cis-Fer-2,17-dGp **9** was analogous to Fer-17-dGp (Figure 8B). This compound was not completely detected in diluted aqueous samples due to extremely low signal intensity (Figure 6).

### 2.9. Extraction of Gomphrenins during Tea Brewing of G. globosa in Aqueous Citric Acid Solutions

In general, lower concentrations of extracted natural gomphrenins were determined during tea brewing in aqueous citric acid solutions in comparison to aqueous solutions. Exploration of the tea brewing data obtained for citric acid solutions revealed similar profiles of betacyanin degradation products to aqueous solutions. Cis-Fer-2,17-dGp **9** (*m/z* 639) previously observed in aqueous solutions was not detected in the citric acid solutions (Figure 6). The greatest concentration of acylated gomphrenins was obtained after 15 min of tea brewing for undiluted and fivefold diluted samples (Figure 5E,F), and their fast decline was observed within the next 15 min. In contrast to aqueous solutions, the degradation of substrates, cis-Fer-Gp/-IGp **5/5′**, Fer-Gp/-IGp **6/6′**, cis-Coum-Gp/-IGp **13/13′**, Coum-Gp/-IGp **14/14′,** and Sin-Gp/-IGp **20/20′** was much faster especially for diluted samples, with no substrates being retained after 60 min of the experiment (Figure 5F). The pair of Gp/-IGp **1/1′** was more labile than acylated gomphrenins, reaching the greatest concentration after 5 min and degrading very quickly during the next 25 min of tea brewing (Figure 5G,H).

### 2.10. Generation of Decarboxylated Derivatives of Gomphrenins in Aqueous Citric Acid Solutions and Their Time-Dependent Concentration Profiles

Similarly to aqueous tea samples, the pairs of diastereomers of Fer-, Coum- and Sin-17-dGp/-IGp (**8/8′**, **16/16′**, **21/21′**, respectively) were detected in citric acid tea infusions. An increase of their concentrations was observed during the first 30 min in the undiluted samples (Figure 8B–D) with a subsequent slight decline, in contrast to their precursors (Figure 5E). A similar effect was observed for diluted samples, however, in that case, the decline was observed after the first 15 min of tea brewing (Figure 8B–D). Interestingly, cis-Fer-17-dGp **7** and cis-Coum-17-dG **15** were detected both in undiluted and diluted citric acid samples, whereas these compounds were not present in diluted aqueous samples (Figure 6). For Fer-17-dGp **8** (Figure 9A) and Coum-17-dGp **16** (Figure 9D), a very strong increase of their time-dependent CR values in all samples extracted in citric acid solutions was observed. An increase of the CR ratio for Sin-17-dGp **21** (Figure 9H) could be observed during the heating of citric acid undiluted samples, whereas in diluted samples, an increase of the ratio was noticed until 30 min, with a subsequent decrease at 60 min. These changes were not observed in aqueous solutions because natural gomphrenins were more stable which resulted in low and steady CR ratios. For 17-dGp **2** (Figure 9F), an increase of the CR ratio was observed during the heating of the diluted samples. Other detected mono-decarboxylated derivatives, **17** and **12**, were assigned to Coum-2-dGp and Fer-15-dGp, respectively. Their concentration profiles were the same as for Coum-17-dGp **16** and Fer-17-dGp **8**, respectively, for undiluted and diluted samples (Figure 8C). For Coum-2-dGp **17** (Figure 9C) and Fer-15-dGp **12** (Figure 9B), very similar signal CR ratios were observed to the CR for Coum-17-dGp **16** (Figure 9D) and Fer-17-dGp **8** (Figure 9F). Fer-2-dIGp **10′** (*m/z* 683) was detected (Table 1), while this compound was not observed in aqueous samples (data not shown). The maximum signal intensity for 15-dGp **12** was observed after 5 min and 15 min of heating in undiluted and diluted aqueous solutions of citric acid, respectively. The Signal of Sin-15-dGp **24** started diminishing after 30 min and 15 min for undiluted and diluted samples, respectively (Figure 8D). The CR ratios obtained for 17-dGp **2** and 2-dGp **4** (Figure 9F and Figure 9E, respectively) increased faster than the ratios for the acylated derivatives for all diluted samples. In contrast, 15-dGp **3** (Figure 9G) was the most labile and was not present in citric acid solutions after 60 min of heating.

### 2.11. Studies on Model Acylated Gomphrenins Isolated from G. globosa Extract

For the aim of selective generation of simplified profiles of acylated gomphrenin derivatives for clear referencing of complex mixtures obtained during the tea brewing study, other heating experiments were performed on purified diastereomers isolated from *G. globosa* extract. These selected pigments were heated for 10–20 min at 90 °C only in aqueous citric acid solutions which were sufficient for providing clear comprehensive profiles of the referential derivatives and enabled finding the absorption maxima for most of the relevant chromatographically studied compounds. In a few cases, some derivatives generated in the model experiments were not detected in the tea brewing products. Selected chromatograms of the reaction mixtures obtained by heating of purified acylated gomphrenins registered at λ 500 nm are depicted in Figure 4C–E.

### 2.12. Identification of Thermally Mono-Decarboxylated Derivatives of Isolated Gomphrenins

Similarly to the tea brewing experiments, heating of the isolated model gomphrenins resulted primarily in the generation of their corresponding 17-decarboxylated derivatives Fer-, Coum-, and Sin-17-dGp/-dIGp (**8/8′**, **16/16′**, **21/21′**). From the 2-decarboxylated derivatives, Fer-2-dGp/-dIGp **10/10′** and Coum-2-dGp/-dIGp **17/17′** were present in great concentrations (Table 1). The pair of **22/22′**, previously not detected in the tea brewing products, was tentatively assigned in the heating products of Sin-Gp/-IGp (**20/20′**) to sinapoyl-2-decarboxy-gomphrenin/-isogomphrenin (Sin-2-dGp/-dIGp), based on the absorption λ_max_ 536 nm and the results of HRMS analyses which confirmed the molecular formula (measured *m/z* 713.2156 vs. calculated 713.2188, Table 2). LCMS-IT-TOF fragmentation yielded three ions of the same molecular formula, as obtained for the fragmentation ions of the isomeric compound Sin-17-dGp **21**. 15-decarboxylated derivatives of all the acylated gomphrenins (Fer-15-dGp **12**, Coum-15-dGp **19**, and Sin-15-dGp **24**) were tentatively detected in the heating products as well (Table 1).

### 2.13. Identification of Thermally Bi-Decarboxylated Derivatives of Isolated Acylated Gomphrenins

LC-DAD-ESI-MS/MS analyses of heating products generated from purified acylated gomphrenins resulted in the detection of a wider variety of bi-decarboxylated betacyanins than during the tea brewing experiments. Chromatographic peaks corresponding to Fer-2,17-dGp/-dIGp **11/11′** were detected based on absorption maximum (λ_max_ 520 nm) and the measured molecular formula of C_32_H_34_N_2_O_12_ (*m/z* 639.2174) by HRMS (Table 2), as well as the fragmentation patterns in the MS^2^ mode which afforded *m/z* signal at 463, indicating detachment of a feruloyl moiety within the glucosyl ring of bi-decarboxylated gomphrenin/isogomphrenin (639 − 463 = 176 Da). Other daughter ions of Fer-2,17-dGp/-dIGp **11/11′** detected at *m/z* 301 and 257 were assigned to bi-decarboxylated betanidin/isobetanidin as a result of further deglycosylation (463 − 301 = 162 Da) as well as to tri-decarboxylated betanidin/isobetanidin (301 − 257 = 44 Da) as a result of decarboxylation at C-15. The analogous pair of diastereomers **18/18′** with λ_max_ 515 nm and *m/z* 609.2085 (Table 2) as well as similar fragmentation was assigned to coumaroyl-2,17-bidecarboxy- -gomphrenin/-isogomphrenin (Coum-2,17-dGp/-dIGp). A very weak signal for a pair of **23/23′** was tentatively assigned to sinapoyl-2,17-decarboxy-gomphrenin/-isogomphrenin (Sin-2,17-dGp/-dIGp) with *m/z* 669. This study revealed a complex variety of decarboxylated derivatives present in tea infusions of violet *G. globosa* dried flowers. In general, citric acid addition exhibited a degradative influence on natural gomphrenins’ stability, mainly during the longer tea brewing process (above 15 min), however, the presence of plant matrix significantly increased the stability of identified betacyanins. Moreover, the acylated gomphrenins exhibited greater stability than gomphrenins **1/1′**.

## 3. Materials and Methods

### 3.1. Plant Material

Dried purple flowers of *Gomphrena globosa* L. were purchased from a China market.

### 3.2. Reagents

Formic acid, acetone, LC-MS grade methanol, and water were received from Sigma Chemical Co. (St. Louis, MO, USA). Citric acid was obtained from POCH (Gliwice, Poland).

### 3.3. Tea Brewing of Purple G. Globosa Flowers

To determine the influence of plant matrix on betacyanins’ stability, two (0.940 g) or two fifths (0.188 g) of flowers were brewing at 90 °C in 6 mL of solvent (demi-water or 1% aqueous citric acid). Heated samples were collected subsequently for LC-DAD-ESI-MS analyses after 5, 10, 15, 30, and 60 min, respectively. All of the experiments were performed in triplicate and standard deviation (SD) was calculated. Previously isolated single diastereomers of acylated gomphrenins were used for additional heating experiments in a citric acid solution to preliminarily determine degradation products.

### 3.4. Preparation of Plant Material for Semi-Preparative Chromatography

The extraction of 1 kg of plant material was performed in a 1% aqueous solution of formic acid (*v/v*). The obtained extract was pumped through the 3 cm layer of a silica gel in a Büchner funnel to separate the solid particles and the suspension. Separation and purification of the extract proceeded onto the ion exchange bed of a strong anion exchanger Sepra^TM^ ZT-SAX with a 30 μm pore size (Phenomenex, Torrance, CA, USA). After adsorption, pigments were eluted by a solution of 5% aqueous formic acid in 50% acetone (*v/v*). The collected eluate was concentrated in a rotary evaporator under reduced pressure at 25 °C and then purified by semi-preparative liquid chromatography.

### 3.5. Semi-Preparative Chromatography

For purification and isolation of gomphrenin pigments from *G. globosa* flowers, a semi-preparative HPLC system with a pump (Knauer HPLC PUMP 64, Knauer, Berlin, Germany), a UV−Vis detector (Knauer), and LP-Chrom operating software, (Lipopharm, Zblewo, Poland) equipped with a C18 (250 × 50 mm i.d., 40 μm) column (Interchim, Montlucon, France) and a 10 × 10 mm guard column (Phenomenex), was applied. Separations were carried out under the following gradient system (System 1) composed of 1% aqueous formic acid (A) and acetone (B) as follows: 0 min, 20% B; increasing linearly to 20 min, 25% B; increasing linearly to 30 min, 30% B; increasing linearly to 40 min, 35% B. The injection volume was 30 mL and the flow rate was 50 mL/min. For further purification of gomphrenin I, the following gradient system (System 2) consisting of 1% aqueous formic acid (A) and acetone (B) was applied: 0 min, 8% B; increasing linearly to 20 min, 10% B; increasing linearly to 30 min, 12% B; increasing linearly to 40 min, 14% B. Purified gomphrenin II and III were obtained using an isocratic preparative HPLC system (System 3) composed of 1% aqueous formic acid (A) and acetone (B) and it was as follows: 0 min, 25% B to 30 min. Finally, for gomphrenin IV purification, the following isocratic system (System 4) composed of 4% aqueous formic acid (A) and acetone (B) was as follows: 15% B for 30 min. Detection was performed at λ 505 nm with a PDA UV/Vis detector.

### 3.6. LC-DAD-ESI-MS/MS Analyses

For qualitative and quantitative analyses of tea infusion samples as well as fractions collected during semi-preparative chromatographic separation, a low resolution LC-MS-8030 mass spectrometric system (Shimadzu, Kyoto, Japan) coupled to LC-20ADXR HPLC pumps, an injector model SIL-20ACXR, and a PDA detector (photodiode array) model SPD-M20A, all controlled with LabSolutions software, version 5.60 SP1 (Shimadzu), was applied. The samples were eluted through a 150 × 4.6 mm i.d., 5.0 μm, Kinetex C18 chromatographic column preceded by a guard column of the same material (Phenomenex). The injection volume was 50 μL and the flow rate was 0.5 mL/min. The column was thermostated at 40 °C. Samples solutions were pumped through the column under the following elution gradient system (System 5) composed of 2% aqueous formic acid (A) and pure methanol (B) as follows: 0 min, 26% B; increasing linearly to 32 min, 90% B; increasing linearly to 35 min, 12% B; increasing linearly to 40 min, 14% B. The column was thermostated at 40 °C. The injection volume was 50 μL and the flow rate was 0.5 mL/min. The detection was performed in the full PDA range and at selected wavelengths (540, 505, 480, and 440 nm). The ionization electrospray source operated in positive mode (ESI+) at an electrospray voltage of 4.5 kV, capillary temperature 250 °C, and using N_2_ as a sheath gas. The LC-MS system was controlled by LabSolutions software, version 5.60 SP1 (Shimadzu), recording total ion chromatograms, mass spectra, and ion chromatograms in selected ion monitoring mode (SIM), as well as the fragmentation spectra. Argon was used as the collision gas for the collision-induced dissociation (CID) experiments. The relative collision energies for MS/MS analyses were set at −35 V.

### 3.7. Chromatographic Analyses with Detection by Ion-Trap Time-of-Flight System (LCMS-IT-TOF)

The mass spectrometer (Shimadzu) with electrospray ionization mode (ESI) coupled to the HPLC Prominence (Shimadzu) was applied to record all mass spectra. The compounds were separated on a 50 × 2.1 mm i.d., 1.9 μm Shim Pack GISS C18 column (Shimadzu) thermostated at 40 °C. Samples were dosed in a volume of 2 μL and the flow rate was 0.2 mL/min. The separation of the analytes was performed in the same gradient systems as in the case of LC-DAD-ESI-MS/MS. The parameters of LCMS-IT-TOF spectrometer were set as follows: curved desolvation line (CDL) and heat block temperature was 230 °C, the nebulizing gas flow rate was 1.5 L/min, and capillary voltage was 4.5 kV. Positive ion mode with the mass range within 100–2000 Da was applied for recording all mass spectra. The collision energy was in the range of 12–50% independence on the structure of the compounds. The Formula Predictor within LCMS Solution software was used for the elaboration of results obtained in high-resolution mass spectrometry experiments (HRMS). Only an empirical formula with a mass error below 5 ppm was considered.

## 4. Conclusions

Herein, it is the first qualitative and quantitative report on gomphrenin derivatives formed during a tea brewing process in aqueous and aqueous citric acid solutions. The presence of gomphrenins in *G. globosa* flower extract may exhibit a positive impact on human health by regular drinking of its tea infusions. The rich profile of decarboxylated gomphrenins may contribute to their antioxidative and other pro-health activities; however, further research on the group of these biologically active compounds is still needed.

## Figures and Tables

**Table 1:** Chromatographic, spectrophotometric, and mass spectrometric data of the analysed natural gomphrenins and their derivatives in *G. globosa* floral extracts after tea brewing at 90 °C.

No.	Compound	Abbreviation	R_t_ [min]	λ_max_ [nm]	*m/z* [M + H]^+^
**1**	gomphrenin	Gp	4.0	538	551
**1′**	isogomphrenin	IGp	4.2	538	551
**2**	17-decarboxy-gomphrenin	17-dGp	4.1	507	507
**2′**	17-decarboxy-isogomphrenin	17-dIGp	4.4	507	507
**3**	15-decarboxy-gomphrenin	15-dGp	4.8	530	507
**4/4′**	2-decarboxy-gomphrenin	2-dGp	5.3	533	507
**5**	cis-feruloyl-gomphrenin	cis-Fer-Gp	11.4	545	727
**5′**	cis-feruloyl-isogomphrenin	cis-Fer-IGp	12.9	545	727
**6**	feruloyl-gomphrenin	Fer-Gp	16.3	545	727
**6′**	feruloyl-isogomphrenin	Fer-IGp	23.5	545	727
**7**	cis-feruloyl-17-decarboxy-gomphrenin	cis-Fer-17-dGp	12.9	515	683
**7ʹ**	cis-feruloyl-17-decarboxy-isogomphrenin	cis-Fer-17-dIGp	15.2	515	683
**8**	feruloyl-17-decarboxy-gomphrenin	Fer-17-dGp	17.5	515	683
**9**	cis-feruloyl-2,17-bidecarboxy-gomphrenin	cis-Fer-2,17-dGp	21.4	509	639
**10**	feruloyl-2-decarboxy-gomphrenin	Fer-2-dGp	21.6	537	683
**8′**	feruloyl-17-decarboxy-isogomphrenin	Fer-17-dIGp	24.0	515	683
**11**	feruloyl-2,17-bidecarboxy-gomphrenin	Fer-2,17-dGp	24.1	520	639
**10′**	feruloyl-2-decarboxy-isogomphrenin	Fer-2-dIGp	27.9	537	683
**12**	feruloyl-15-decarboxy-gomphrenin	Fer-15-dGp	30.4	530	683
**11′**	feruloyl-2,17-bidecarboxy-isogomphrenin	Fer-2,17-dIGp	31.0	520	639
**13**	cis-coumaroyl-gomphrenin	cis-Coum-Gp	11.2	545	697
**13′**	cis-coumaroyl-isogomphrenin	cis-Coum-IGp	12.8	545	697
**14**	coumaroyl-gomphrenin	Coum-Gp	16.1	545	697
**14′**	coumaroyl-isogomphrenin	Coum-IGp	21.5	545	697
**15**	cis-coumaroyl-17-decarboxy-gomphrenin	cis-Coum-17-dGp	12.9	512	653
**15′**	cis-coumaroyl-17-decarboxy-isogomphrenin	cis-Coum-17-dIGp	15.0	512	653
**16**	coumaroyl-17-decarboxy-gomphrenin	Coum-17-dGp	17.4	512	653
**17**	coumaroyl-2-decarboxy-gomphrenin	Coum-2-dGp	22.5	539	653
**16′**	coumaroyl-17-decarboxy-isogomphrenin	Coum-17-dIGp	22.8	512	653
**18**	coumaroyl-2,17-bidecarboxy-gomphrenin	Coum-2,17-dGp	26.1	515	609
**17′**	coumaroyl-2-decarboxy-isogomphrenin	Coum-2-dIGp	28.8	539	653
**19**	coumaroyl-15-decarboxy-gomphrenin	Coum-15-dGp	31.0	532	653
**18′**	coumaroyl-2,17-bidecarboxy-isogomphrenin	Coum-2,17-dIGp	31.9	515	609
**20**	sinapoyl-gomphrenin	Sin-Gp	15.4	545	757
**20′**	sinapoyl-isogomphrenin	Sin-IGp	23.6	545	757
**21**	sinapoyl-17-decarboxy-gomphrenin	Sin-17-dGp	17.1	517	713
**22**	sinapoyl-2-decarboxy-gomphrenin	Sin-2-dGp	19.3	536	713
**23**	sinapoyl-2,17-bidecarboxy-gomphrenin ^a^	Sin-2,17-dGp	21.6	- ^b^	669
**23′**	sinapoyl-2,17-bidecarboxy-isogomphrenin ^a^	Sin-2,17-dIGp	22.4	- ^b^	669
**21′**	sinapoyl-17-decarboxy-isogomphrenin	Sin-17-dIGp	24.0	517	713
**22′**	sinapoyl-2-decarboxy-isogomphrenin	Sin-2-dIGp	26.2	536	713
**24**	sinapoyl-15-decarboxy-gomphrenin ^a^	Sin-15-dGp	29.7	- ^b^	713

^a^—Tentatively identified. ^b^—Due to coelution with impurities, observation of λ_max_ was not possible.

**Table 2:** High-resolution mass spectrometric data obtained by LCMS-IT-TOF measurements of purified acylated gomphrenins isolated from *G. globosa* flowers and their derivatives generated after heating of acylated gomphrenins at 90 °C in aqueous citric acid solutions.

No.	Compound	Molecular Formula	[M + H]^+^ Observed	[M + H]^+^ Predicted	Error [mDa]	Error [ppm]	Principal MS^2^ Ions
**5**	cis-Fer-Gp	C_34_H_35_N_2_O_16_	727.1947	727.1981	−3.4	−4.68	551; 389; 345
**6**	Fer-Gp	C_34_H_35_N_2_O_16_	727.1970	727.1981	−1.1	−1.51	551; 389; 345
**7**	cis-Fer-17-dGp	C_33_H_35_N_2_O_14_	683.2068	683.2083	−1.5	−2.20	507; 345
**8**	Fer-17-dGp	C_33_H_35_N_2_O_14_	683.2081	683.2083	−0.2	−0.29	507; 345; 301
**10**	Fer-2-dGp	C_33_H_35_N_2_O_14_	683.2075	683.2083	−0.8	−1.17	507; 345
**11**	Fer-2,17-dGp	C_32_H_35_N_2_O_12_	639.2174	639.2185	−1.1	−1.72	463; 301, 257
**12**	Fer-15-dGp	C_33_H_35_N_2_O_14_	683.2063	683.2083	−2.0	−2.93	507; 345; 301
**13**	cis-Coum-Gp	C_33_H_33_N_2_O_15_	697.1886	697.1875	1.1	1.58	551; 389
**14**	Coum-Gp	C_33_H_33_N_2_O_15_	697.1887	697.1875	1.2	1.72	551; 389; 345
**15**	cis-Coum-17-dGp	C_32_H_33_N_2_O_13_	653.1960	653.1977	−1.7	−2.60	507; 345
**16**	Coum-17-dGp	C_32_H_33_N_2_O_13_	653.1990	653.1977	1.3	1.99	507; 345; 301
**18**	Coum-2,17-dGp	C_31_H_33_N_2_O_11_	609.2085	609.2079	0.6	0.98	463; 301; 257
**20**	Sin-Gp	C_35_H_37_N_2_O_17_	757.2117	757.2087	3.0	3.96	551; 389; 345; 301
**21**	Sin-17-dGp	C_34_H_37_N_2_O_15_	713.2205	713.2188	1.7	2.38	507; 345; 301
**22**	Sin-2-dGp	C_34_H_37_N_2_O_15_	713.2156	713.2188	−3.2	−4.49	507; 345; 301
